# Experimental Evaluation of Shear Behavior of Stone Masonry Wall

**DOI:** 10.3390/ma14092313

**Published:** 2021-04-29

**Authors:** Maria Luisa Beconcini, Pietro Croce, Paolo Formichi, Filippo Landi, Benedetta Puccini

**Affiliations:** Department of Civil and Industrial Engineering—Structural Division, University of Pisa, 56122 Pisa, Italy; ml.beconcini@ing.unipi.it (M.L.B.); p.formichi@ing.unipi.it (P.F.); benedetta.puccini@ing.unipi.it (B.P.)

**Keywords:** masonry, masonry mechanical parameters, flat jack test, shear test, shear behavior, capacity curve, seismic vulnerability

## Abstract

The evaluation of the shear behavior of masonry walls is a first fundamental step for the assessment of existing masonry structures in seismic zones. However, due to the complexity of modelling experimental behavior and the wide variety of masonry types characterizing historical structures, the definition of masonry’s mechanical behavior is still a critical issue. Since the possibility to perform in situ tests is very limited and often conflicting with the needs of preservation, the characterization of shear masonry behavior is generally based on reference values of mechanical properties provided in modern structural codes for recurrent masonry categories. In the paper, a combined test procedure for the experimental characterization of masonry mechanical parameters and the assessment of the shear behavior of masonry walls is presented together with the experimental results obtained on three stone masonry walls. The procedure consists of a combination of three different in situ tests to be performed on the investigated wall. First, a single flat jack test is executed to derive the normal compressive stress acting on the wall. Then a double flat jack test is carried out to estimate the elastic modulus. Finally, the proposed shear test is performed to derive the capacity curve and to estimate the shear modulus and the shear strength. The first results obtained in the experimental campaign carried out by the authors confirm the capability of the proposed methodology to assess the masonry mechanical parameters, reducing the uncertainty affecting the definition of capacity curves of walls and consequently the evaluation of seismic vulnerability of the investigated buildings.

## 1. Introduction

Masonry buildings are a large part of a built environment, especially in historical towns. Since these structures were mostly built according to empirical rules and without following specific seismic design provisions, they are often characterized by a high seismic vulnerability. In addition, the need of preservation makes the assessment of their structural performance a significant criterion for planning maintenance and strengthening interventions [1,2].

Understanding and modelling the mechanical behavior of masonry elements is a long-standing challenge in civil engineering [3]. In fact, the characterization of mechanical properties of masonry is certainly one of the most delicate issues in the process, especially in the field of heritage structures, where modern studies suggest also to resort to advanced probabilistic approaches [4].

The characterization of the shear behavior of each wall is the basis for a sound assessment of the seismic vulnerability of a masonry building. As known, seismic performance, which is commonly evaluated through nonlinear static analysis, is highly dependent on assumptions related to the main mechanical parameters of the material: shear modulus, G; elastic modulus, E; shear strength, $\tau_{k}$; global stiffness; and displacement capacity. Sensitivity studies carried out by the authors [1,5,6] emphasized that the seismic risk index $I_{R}$, which summarizes the seismic performance of the structure, could significantly vary depending on the assumptions about the shear behavior of masonry walls.

As confirmed by the available experimental results [7], the estimate of the shear modulus of the masonry is characterized by huge uncertainty. Actually, the shear modulus and the effective stiffness of masonry walls not only are a function of the masonry type, but significantly depend on the adopted test setup [8], which usually can include vertical compression tests [9], diagonal compression tests [10], and shear compression tests [11]. Since global resistance, effective stiffness, and shear failure mechanisms of the wall depend on compressive stresses, quality of mortar [8,12], and other influencing parameters, the shear behavior of masonry walls has been deeply investigated [2,7,8,13,14,15,16,17,18] to determine the most relevant relationships.

It must be remarked that in situ test procedures usually consist of diagonal compression and shear compression tests performed on panels, suitably obtained by cutting the walls, which are completely reconstructed after the test. Owing to the fact that this kind of tests can be extremely invasive, the setup of new testing methodologies allowing suitably fast but accurate estimation of masonry parameters is thus a priority, also in view of the definition of the capacity curves of masonry walls [5].

In the framework of an important research project regarding the seismic assessment of more than 80 school masonry buildings in Florence, a wide in situ experimental campaign was carried out by the authors on different masonry typologies [2]. The experimental results, compared with the ones derived adopting different test methods on similar masonry walls, allowed for identifying suitable modifications of the test arrangements.

In the paper, an innovative in situ shear test arrangement for masonry panels is illustrated, together with the first promising experimental results. The proposed test procedure, which can be considered less invasive than the alternatives, is able to provide the relevant parameters that are needed to model the shear behavior of walls (i.e., elastic modulus, E; shear modulus, G; and shear strength, $\tau_{k}$) [1,2].

In the following sections, the current procedures for the mechanical characterization of masonry walls are preliminarily recalled and commented. Then the proposed testing methodology is presented, also referring to the experimental results.

Finally, the obtained results are critically discussed, highlighting the most significant parameters, also considering their dependence on basic assumptions and the main features of other commonly adopted shear models.

## 2. Mechanical Behavior of Masonry Walls

### 2.1. Masonry Categories

Masonry has been the main construction material for millennia. Since historic constructions, built by craftspeople, are not the result of industrial processes, they are characterized by a large variety of masonry types.

The quality and mechanical properties of a given masonry type depend on many factors, such as availability of the constituent materials, building importance, skills of workmanship, local customs, and architectural canons, if any [2]. For that reason, the properties of similar existing masonry can vary in a wide range.

Some preliminary guidance can be often found in modern structural codes, where typical ranges of values for compressive resistance, $f_{m}$; shear resistance, $\tau_{k}$; elastic modulus, E; shear modulus, G; and specific weight, γ, are given for recurrent masonry typologies. For example, the Italian Building Code provides information for eight recurrent masonry typologies: irregular stone masonry, partially dressed stone masonry, well-dressed stone masonry, irregular soft-stone masonry, squared soft-stone masonry, squared hard-stone masonry, brickwork masonry with lime-based mortar, and hollow-bricks masonry with cement mortar [19]. These ranges, summarized in Table 1, refer to low-quality masonry, but they can be suitably adjusted and increased for masonry of improved quality, depending on mortar strength, thickness of mortar joints, regularity of courses, efficiency of transverse connections, and quality of inner core if any, as well as on strengthening intervention, like grout injection and external concrete layers.

Anyhow, even if the masonry typology is well identified, subjective judgements can notably influence the estimation of mechanical properties. In practical cases, a possible solution could consist in supplementing the reference values of the mechanical properties, pertaining to the identified masonry typology, with the results of limited semidestructive or nondestructive in situ tests. A procedure for the identification of masonry classes and their mechanical parameters is presented in [2], based on the analysis of available datasets of test results, complemented with some additional information on masonry quality [18] and in situ test results.

### 2.2. Database of Material Test Results

In the framework of already-cited vulnerability assessments performed by the authors during the last 4 years on about 80 masonry school buildings in the Municipality of Florence, a large experimental campaign, consisting of in situ and laboratory tests, was carried out [2,6]. The experimental results, supplemented with relevant literature data, allowed for the setup of a consistent database of mechanical properties of various masonry types, from which it was possible to derive a rational classification. At the same time, adopting a general procedure, based on cluster analysis of raw data, successfully proposed by the authors to identify homogenous populations within large dataset [20], sound information about statistical properties of relevant investigated mechanical parameters was obtained.

The database mainly contains results of about 100 double flat jack tests performed on the most common masonry typologies by three different laboratories, according to ASTM standards [9]. More precisely, 67 tests concern stone masonry walls, 26 tests concern solid brick masonry, and 4 tests concern other masonry types.

As said, considering the already-mentioned guidelines for the application of the Italian Building Code [19], as well as the so-called masonry quality index (MQI) [18] derived by visual inspection, the collected results allowed for identifying the most relevant masonry classes and the statistical parameters characterizing their main mechanical properties: compressive strength, $f_{m}$; elastic modulus, E; and shear modulus, G, [2], which are necessary to calibrate the partial safety factors for resistance [21].

The relevant statistical parameters obtained are summarized in Table 2. In the table, mean values and coefficients of variations (COVs) of compressive strength, elastic modulus, and shear modulus are given not only for the whole data collection, but also for each homogenous population/class, identified according to the previously mentioned procedure [2,20]. The three classes refer to low-, medium-, and high-quality masonry; this classification, simple and very effective from the engineering point of view, reflects the results of the cluster analysis [2].

Elastic and shear moduli have been derived linearizing the experimental σ−ε diagrams in three stress ranges to describe the relevant sections of material behavior (i.e., $\left[0.1\;\sigma_{\max}–0.4\;\sigma_{\max}\right]$, $\left[0.4\;\sigma_{\max}–0.7\;\sigma_{\max}\right]$, and $\left[0.7\;\sigma_{\max}–\sigma_{\max}\right]$). In Table 2, results are reported only for the first section, $\left[0.1\;\sigma_{\max}–0.4\;\sigma_{\max}\right]$, corresponding to the quasi-elastic branch. For a complete description of the methodology for masonry class identification and an overview of most relevant results, the interested reader can refer to [2].

Figures in Table 2 demonstrate that, as expected, the identification of homogenous populations is an essential step of the analysis for a sound estimation of relevant statistical parameters: in fact, that elaboration of the whole dataset leads to very rough results.

Notwithstanding the advances in masonry class identification and the reduction of uncertainties regarding the associated mechanical parameters, reliable information about shear strength $\tau_{k}$ is still lacking. For that reason, $\tau_{k}$ is currently estimated by means of suitable correlation expressions from rough reference data, like those given in Table 1, or, indirectly, from other mechanical properties. For example, based on experimental data provided in the relevant literature, a suitable expression has been proposed in [7], expressing $\tau_{k}$ as a function of the shear modulus G:

(1) $$G\approx 1500–2000\;\tau_{k}.$$

### 2.3. Shear Behavior of Masonry Walls

Shear behavior of masonry, first evaluated in situ by Sheppard [11], is currently investigated according to different test arrangements. Usually, tests are performed on masonry panels, isolated from the remaining part of the masonry wall by suitable cutting tools, like diamond wires or circular saws.

**Figure 1:**
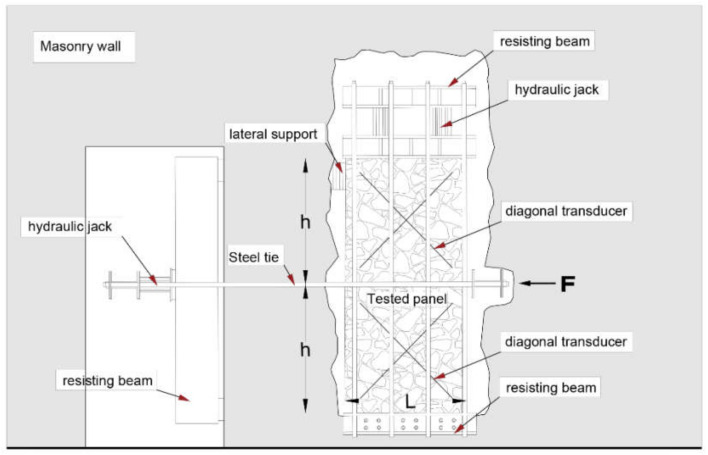
Typical test arrangement for shear compression tests.

**Figure 2:**
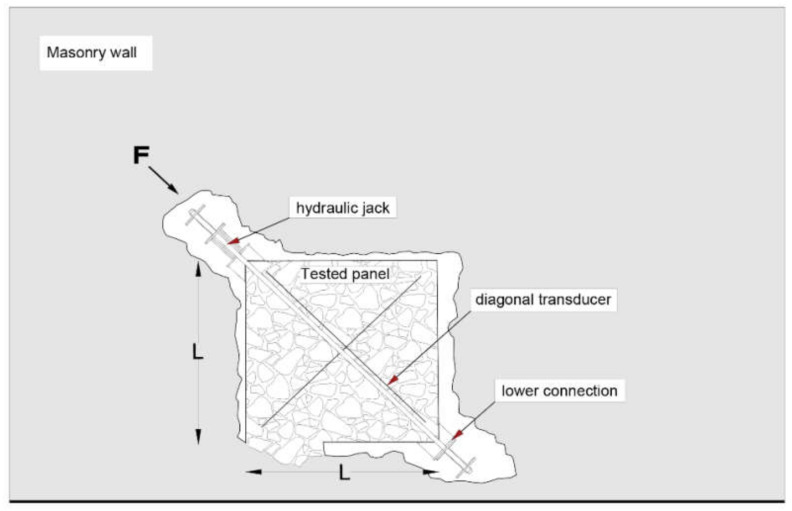
Typical test arrangement for in situ diagonal compression test of masonry panels.

The mainly adopted test arrangements are illustrated in Figure 1 and Figure 2. They basically include shear compression tests (Figure 1) and diagonal compression tests (Figure 2). Clearly, shear characteristics can be derived from compression tests only by means of indirect empirical relationships [7].

Shear compression tests are carried out on rectangular panels, whose height, 2 h, is approximately twice the base length, L, so that the median of the height divides the whole panel in two square parts. In the original Sheppard scheme [11], the panel, separated from the remaining part only by vertical cut(s), is subjected to the actual vertical loads; therefore, the real value of the compressive stress $\sigma_{0}$ needs to be measured or calculated. In the modified scheme illustrated in Figure 1, the panel is separated from the remainder of the wall also on the upper part by a horizontal cavity: the compressive stress $\sigma_{0}$ is thus applied, and maintained constant during the test, by means of hydraulic jacks resisted by a suitable system of steel ties and steel profiles.

During the test, an increasing horizontal force *F* is applied at the mid height of the panel by means of a steel metal profile actuated by a hydraulic jack via two steel bars, inducing a significant shear stress pattern in the panel.

Diagonal displacements are measured via eight transducers, placed on both sides of the sample along the diagonals of the two square panels. Horizontal displacements and any possible rotations are measured by means of six additional transducers, duly positioned along each side of one vertical edge of the panel (at the base, at the center, and on top).

During the test, suitable force-displacement curves are plotted. The shear strength of the masonry, $\tau_{k}$, is thus derived according to the well-known Turnšek and Ĉaĉoviĉ criterion for shear failure [22]:

(2) $$V_{\max}=0.5\;F_{\max}=\frac{1.5}{b}A\;\tau_{k}\sqrt{1+\frac{\sigma_{0}}{1.5\tau_{k}},}$$

as a function of the maximum applied force, *A* being the cross-sectional area of the panel. In Equation (2), the shear stress distribution factor, b, which accounts for the actual variability of the shear stresses in the cross section [23], can be set to $b=\min\;\left(1.5;\;2\;h/L\right)$.

Diagonal compression tests are carried out on a square panel isolated from the masonry wall and anchored only through a part of the lower horizontal edge [10,24] (Figure 2). The compressive diagonal force A is applied by a hydraulic jack, placed on one of the two corners. The panel is equipped with four or more displacement transducers, arranged along the diagonals on both sides, allowing for the measurement of the deformations at each load step. In the regular test procedure, couples of equal cycles of loading and unloading, with a load increase of 10 kN at each step, are applied till failure. Hence, the shear strength is evaluated as:

(3) $$\tau_{k}=\frac{\sqrt{2}}{2}\frac{F_{\max}}{A_{n}},$$

where $A_{n}$ is the net area of the panel, determined as the average of the width and height of the specimen multiplied by its thickness [25].

A review of test procedures and relevant results reported in the literature can be found in [7], while more details about in situ diagonal test and shear compression tests are reported in [14,15], respectively.

**Figure 3:**
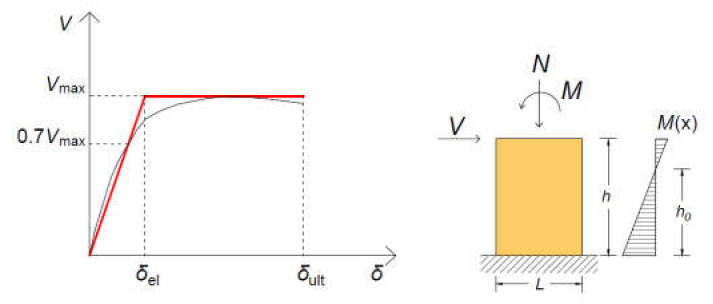
Modelling the shear behavior of masonry panels via bilinear approximation.

The shear force-displacement behavior obtained from experimental tests is generally approximated by a bilinear envelope [16,26] (see Figure 3). Let be $V_{\max}$ the maximum shear force measured during the test, the effective stiffness $k_{ef}$ is given by the secant stiffness at $0.7\;V_{\max}$, while the ultimate displacement $\delta_{\mathrm{ult}}$ is defined as the value corresponding to a significant drop ΔV of the descending part of the experimental V−δ curve, for example, $\Delta V=0.2\;V_{\max}$, or as the largest drift reached during the test, if such a large drop ΔV is not attained [16].

The bilinear approximation represents the so-called capacity curve, which is generally adopted to model the nonlinear behavior of masonry walls in seismic analysis [27,28,29,30]. Masonry panels are modelled as Timoshenko’s beams characterized by the stiffness

(4) $$k_{ef}={\left(\frac{h^{2}\left(h_{0}-\frac{h}{3}\right)}{2EJ}+\frac{1.2\;h}{GA}\right)}^{-1},$$

where h is the height of the panel, $h_{0}$ is the shear span, A is the area of the gross section, J is the moment of inertia of the gross section, and E and G are the elastic and shear modulus, respectively.

According to EN1998-1 [31], the effective stiffness should duly consider the influence of cracking. In the absence of more accurate information, it is suggested that the effective stiffness is estimated as one half of the gross sectional stiffness. From the measured effective stiffness, the corresponding elastic and shear modulus can be obtained from Equation (3) suitably fixing the G/E ratio [16,26].

## 3. A Novel Proposal for In Situ Evaluation of Shear Behavior

As known, shear compression tests, carried out according to the previously illustrated scheme in Figure 1, and diagonal compression tests are not only extremely invasive, but also very difficult and complicated to be carried out in practice. In fact, for safety reasons, these test schemes often require, especially when performed in significantly loaded walls, very demanding temporary defense structures. For that reason, an improved alternative method for in situ shear tests is proposed here, aiming to simplify the preparation of the wall panel and to limit the alterations of the existing structure, thus speeding up not only the setup of the specimen, but also the local repairs after the test.

### 3.1. Test Arrangement

The proposed procedure combines a single flat jack test, a double flat jack test, and a suitably modified shear compression test on a properly selected wall panel.

The tested panel is isolated from the remainder of the wall by vertical cuts only, like in the original Sheppard test [11], so that the actual normal stress pattern on the panel remains practically unaltered and can be assessed by a single flat jack test, performed near the tested panel.

The horizontal load is applied to the panel by a hydraulic jack, which is resisted on the opposite side by the remaining part of the structure.

A preliminary in situ investigation phase is devoted to identifying the panel(s) to be tested. The location of the panel is chosen according to the following requirements:

1. Easiness of preparation: identifying existent openings, like doors or windows, the number of vertical cuts needed to define the panel is limited.
2. Adequate carrying capacity of the remainder of the structure: the preparation of a dedicated supporting structure to withstand the horizontal load $F_{\max}$ causing the panel failure is avoided.
3. Significant values of normal stress $\sigma_{0}$ due to vertical loads: the application of external vertical forces is unnecessary.

**Figure 4:**
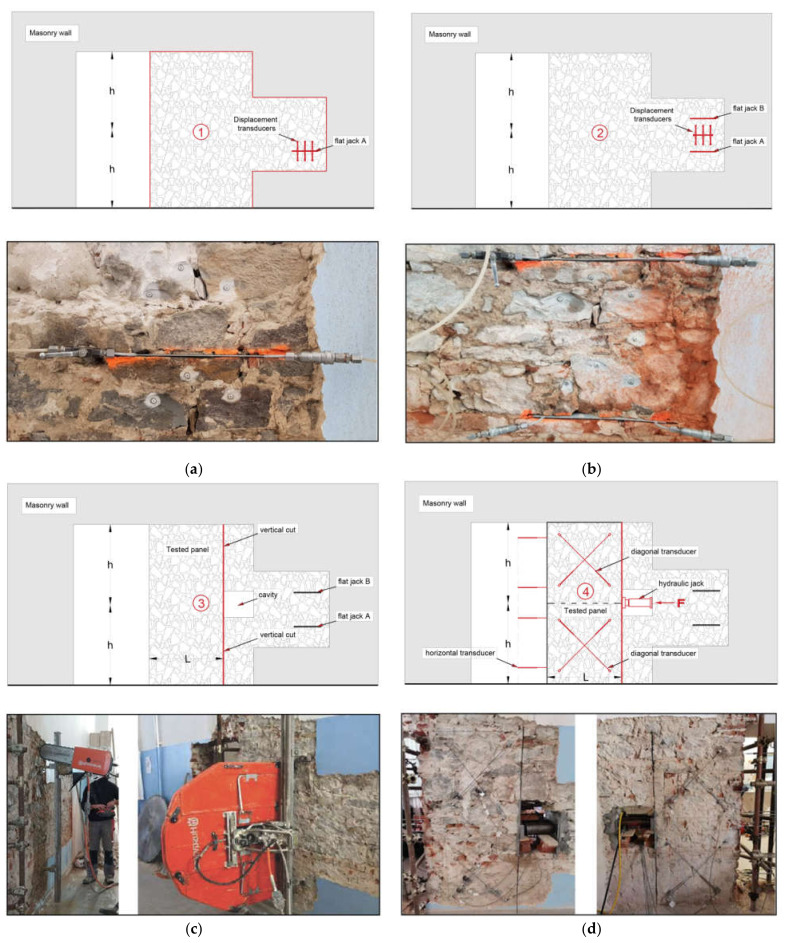
Sequence of relevant test phases: phase 1—removal of the plaster and performance of a single flat jack test (**a**), phase 2—preparation and performance of a double flat jack test (**b**), phase 3—realization of the vertical cut and making of a cavity in the wall to insert the hydraulic jack (**c**), phase 4—setup of the shear test with the placement of the measuring devices, horizontal and diagonal transducers (**d**).

**Figure 5:**
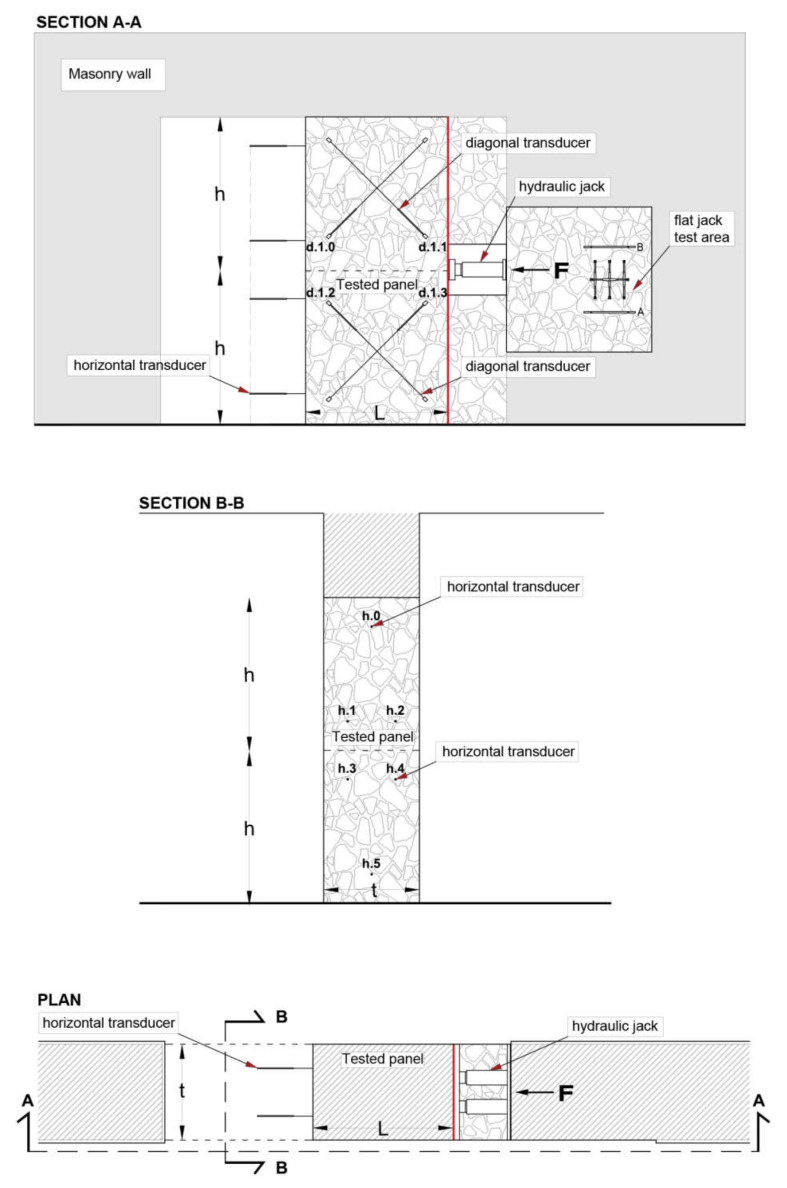
Proposed test arrangement for the evaluation of the shear behavior of masonry panels of length L, height 2 h and thickness t subjected to the horizonal load F.

Once the masonry wall is identified and the plaster removed (if present), the in situ tests are carried out in the following sequence (see Figure 4):

- First, a single flat jack test is carried out to evaluate the actual value of the compressive stress in the wall panel, $\sigma_{0}$. The test is performed near the investigated panel according to the standardized procedure [32,33] (see Figure 4a). Three pairs of gauge points are fixed in the area across the selected cut line, and the initial distances between each pair are read using removable millesimal deformometers. Subsequently, a horizontal cut reproducing the shape and dimensions of the flat jack is made, taking care to minimize the disturbance in the surrounding masonry, and a second set of measurements is taken to determine the strain variation. The flat jack is thus inserted, and pressure is applied till the strain is returned to the original state (i.e., before the cut was made). The corresponding stress $\sigma_{0}$ is thus derived from the pressure required to restore the original masonry conditions [33].
- It is important to recall here that even if a single flat jack test represents a common and powerful tool for the evaluation of the acting stress [34], a careful interpretation of results is required. In fact, conflicting information can be obtained by the different deformometers due to the local presence of inelastic deformations, which is caused by the nonuniform stress distribution after the horizontal cut. An accurate visual inspection of the investigated area around the cut is thus an essential step for a correct understanding of the response of the measuring devices, and a correction may be needed to determine the stress $\sigma_{0}$ as described in [35]. Moreover, since the location of the test is close to the investigated panel but still not coincident (see Figure 4), the resulting stress $\sigma_{0}$ should also be checked and compared with the vertical load computed by load analysis considering the actual loads on the structure.
- After that, a second horizontal cut is made, vertically spaced about 500 mm from the previous one, and a double flat jack test [9] (see Figure 4b) is carried out to determine the elastic modulus, E, and the compressive strength, $f_{m}$. Once the second flat jack is inserted, four pairs of measurement bases, three orthogonal to the cuts and one parallel to them, are installed between the two jacks, and the initial base lengths are measured. The pressure is gradually increased, recording the strain variations for each pressure increment. The test is stopped when the ratio between the increase of jack pressure and the strain increment rapidly drops and the first cracks occur in the masonry. From the experimental σ−ε diagram recorded, the elastic modulus, E, in different behavioral conditions can be derived, as a function of the load level. As already mentioned in Section 2, three different stress ranges are considered: $\left[0.1\;\sigma_{\max}–0.4\;\sigma_{\max}\right]$, $\left[0.4\;\sigma_{\max}–0.7\;\sigma_{\max}\right]$, and $\left[0.7\;\sigma_{\max}–\sigma_{\max}\right]$, representing the quasi-elastic, the cracked, and the plastic sections of the diagram, respectively, so that the pertinent secant elastic moduli can be derived.
- Finally, the shear compression test is performed, as described below (see also Figure 4c,d and Figure 5), to evaluate the shear behavior and to identify the shear strength, $\tau_{k},$ and the shear modulus, G, of the masonry.

The sequence of the relevant phases for the preparation of the tests and their performance is summarized in Figure 4: removal of the plaster and performance of a single flat jack test (phase 1), preparation and performance of a double flat jack test (phase 2), realization of the vertical cut and making of a cavity in the wall to insert the hydraulic jack (phase 3), and setup of the shear test with the placement of the measuring devices, horizontal and diagonal transducers (phase 4).

As anticipated, the test setup is conceived in such a way to simplify the preparation of the wall panel and to facilitate the analysis and interpretation of the behavior of the wall panel itself. To isolate a masonry panel of adequate length, a 1.6–1.8 m long vertical cut is made in the wall at 0.8–0.9 m distance from an opening in such a way to obtain a panel with a height/length ratio of about 2 (see Figure 5). Evidently, the proposed scheme is much more general; in fact, when openings are not present, it remains feasible, although a little more complex, since it requires an additional vertical cut.

As already remarked, in that way the test procedures are simplified and the perturbations of the existing stress pattern in the panel are minimized. In fact, since the wall panel is subjected to an almost constant compressive stress $\sigma_{0}$ caused by the existing vertical loads acting on the structure, the system of steel plates, rods, and hydraulic jacks required in the classical shear compression test scheme (Figure 1) [11] for the introduction of compressive force is no longer necessary.

During the test, an incremental horizontal load is applied at the middle height of the panel by means of two hydraulic jacks, placed in a prismatic cavity of suitable dimensions, previously made in the remainder of the wall. A thick steel plate placed between the hydraulic jacks and the test element allows for obtaining an approximately uniform distribution of the applied pressure.

Considering that the panel is clamped to the remaining part of the structure at both ends and that the load is applied at the middle of the panel, it is assumed that the two approximately square subpanels are subjected to nearly symmetrical shear and compression forces. Different boundary conditions for the upper and lower parts of the panel may cause a lack of symmetry in shear distribution between the two halves of the panel, which should be considered in the analysis of the results [13].

During the test, the horizontal load is gradually applied with intermediate unloading steps, until diagonal cracks occur in one or both the two subpanels, and the maximum load value is thus recorded. Deformations are continuously monitored during the test by a set of 14 linear variable displacement transducer (LVDT) inductive sensors. Eight LVDTs, placed along the diagonals of the two opposite faces of the subpanels, measure the corresponding relative displacements, while the remaining six transducers, placed along the free lateral side, with a symmetrical arrangement with respect to its vertical centerline, measure horizontal displacements. The test arrangement with the location of measuring devices is shown in Figure 5.

**Figure 6:**
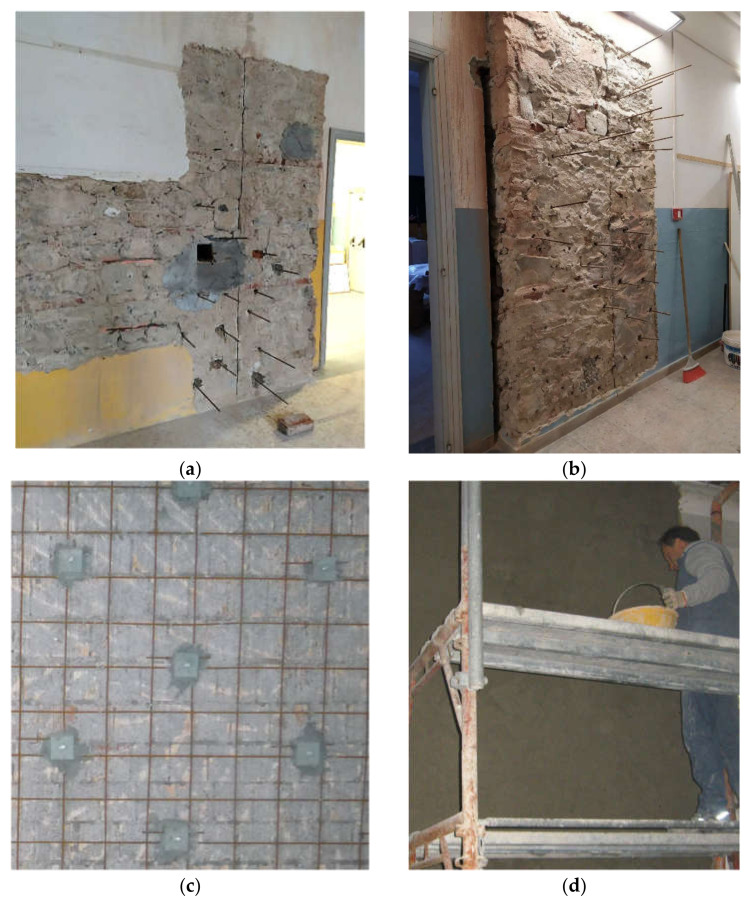
Strengthened technique adopted to restore the tested walls: repair of cut and cavity (**a**), insertion of threaded or reinforcing steel bars (**b**), application of the reinforcing steel mesh (**c**), and final grouting of the concrete layer (**d**).

Once the test is concluded, the panel can be easily repaired and suitably strengthened, adopting appropriate techniques, like addition of steel plates or reinforced concrete exterior layers, duly connected to the masonry. An example is given in Figure 6, where they are represented by the restoring phases of the wall: repair of cut and cavity (Figure 6a), insertion of threaded or reinforcing steel bars (Figure 6a,b), application of the reinforcing steel mesh (Figure 6c), and final grouting of the concrete layer (Figure 6d). If necessary, a more effective strengthening technique can include the insertion of additional steel plates on both sides of the panel.

### 3.2. Results

The first results obtained from the proposed test procedure are illustrated in this section for three stone masonry panels tested during the already-cited experimental campaign carried out by the authors. The proposed methodology is general and can be easily extended also to a different masonry typology. In fact, a test has already been carried out also on solid brick masonry, but results will be presented only for the masonry panels of the same typology.

The stone masonry panels belong to three different buildings, built in Florence at the beginning of the 19th century:

- Panel A is located in the three-story masonry building housing the kindergarten and primary school “Giotto,”
- Panel B is located in the four-story masonry building of the primary school “Cairoli,” and
- Panel C is located in the four-story masonry building of the primary school “Niccolini.”

**Figure 7:**
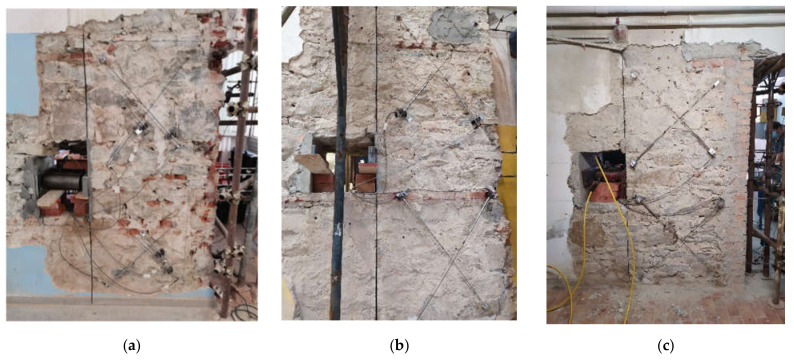
Tested stone masonry panels, Giotto (**a**), Cairoli (**b**), and Niccolini (**c**).

The three masonry panels are shown in Figure 7, while the main characteristics (height, h; length, L; and thickness, t; of the panel) are summarized in Table 3.

The three combined tests (single, double flat jack, and shear tests) are carried out as described in the previous subsection, and the main results are reported in Table 3. More precisely, in the table they are reported: the actual compressive stress on the wall $\sigma_{0}$ derived by the single flat jack test, the compressive strength $f_{m}$, and the secant elastic moduli ($E_{10-40}$, $E_{40-70}$, and $E_{70-100}$) evaluated considering the double flat jack test results, in the pertinent stress range.

**Figure 8:**
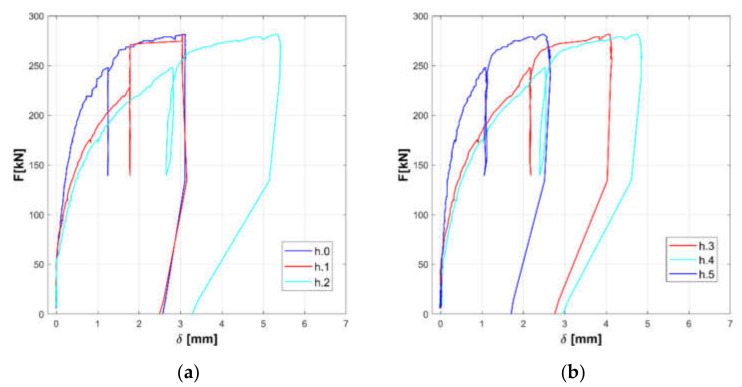
Experimental horizontal displacements of panel A: upper part (**a**) and lower part (**b**).

**Figure 9:**
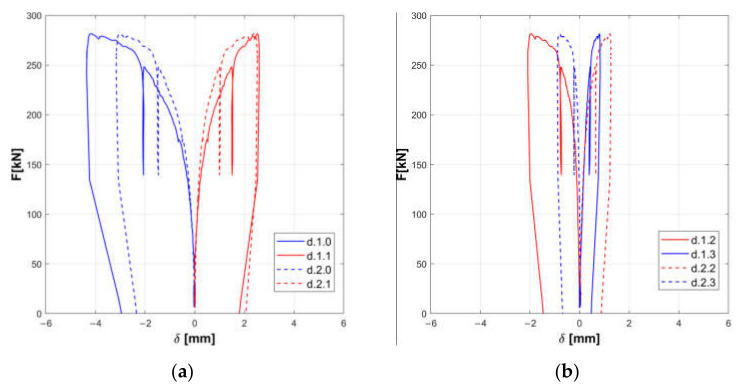
Experimental diagonal displacements of panel A: upper part (**a**) and lower part (**b**).

**Figure 10:**
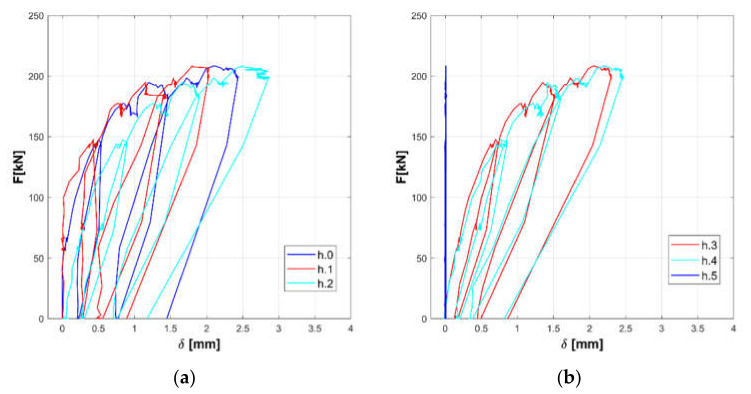
Experimental horizontal displacements of panel B: upper part (**a**) and lower part (**b**).

**Figure 11:**
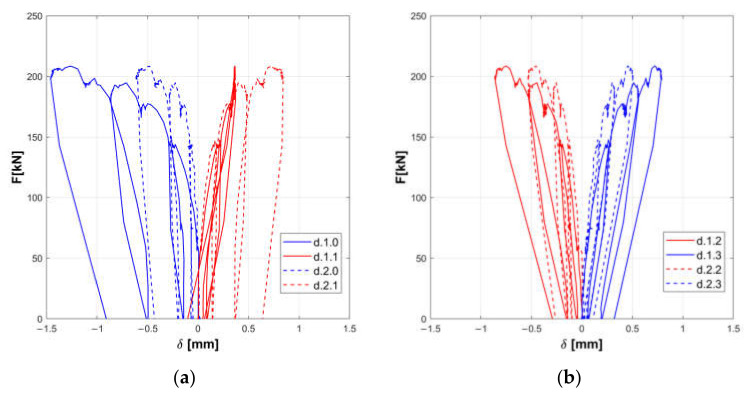
Experimental diagonal displacements of panel B: upper part (**a**) and lower part (**b**).

**Figure 12:**
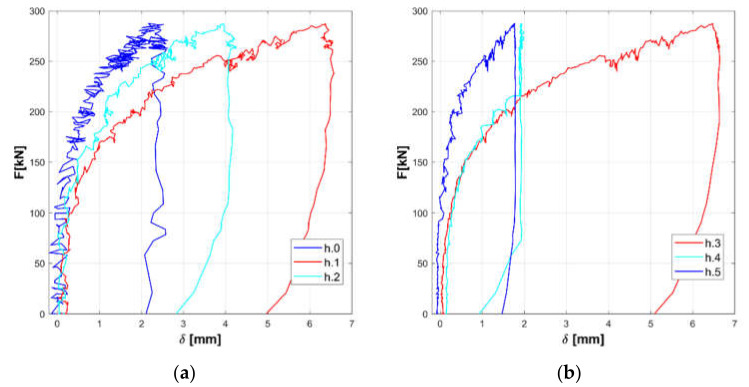
Experimental horizontal displacements of panel C: upper part (**a**) and lower part (**b**).

**Figure 13:**
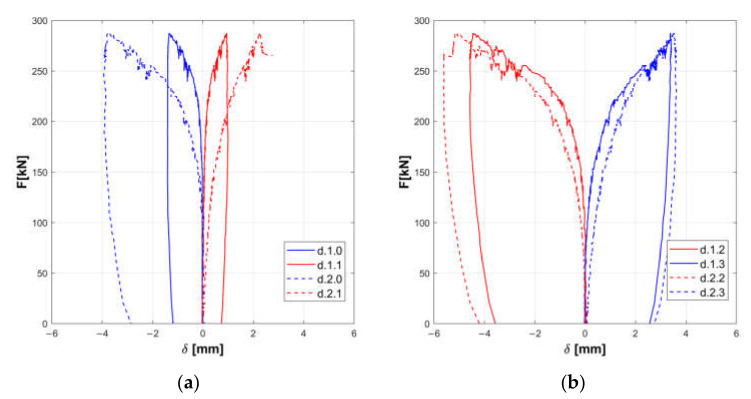
Experimental diagonal displacements of panel C: upper part (**a**) and lower part (**b**).

The responses of the tested panels A, B, and C are summarized in the Figure 8, Figure 9, Figure 10, Figure 11, Figure 12 and Figure 13.

Horizontal displacements are shown in Figure 8, Figure 10 and Figure 12 for the masonry panels A, B, and C, respectively. Diagonal displacements are illustrated in Figure 9, Figure 11 and Figure 13 for masonry panels A, B and C, respectively. In each figure, the results of the shear test are reported considering the total applied load F and the displacements measured by horizontal (h.0, h.1, h.2, h.3, h.4, and h.5 in Figure 5) and diagonal transducers (d.1.0, d.1.1, d.1.2, and d.1.3 in Figure 5 and the corresponding ones placed on the other side of the panel d.2.0, d.2.1, d.2.2, and d.2.3).

To facilitate the comparison, diagrams in frames (a) of the figures refer to the upper part of the investigated panel, while those in frames (b) refer to the lower part.

It must be remarked that some transducers may go out of service during the test: it is the case of h.5 for panel B, which was not working at all; h.4 for panel C in the final stage of the loading phase; and d.2.1 for panel C in the unloading phase.

Looking at the horizontal displacements, it is evident that, generally, the behavior of the two subpanels is similar, provided that the transducers that stop working are disregarded. This outcome validates the assumption that the two subpanels are working in a quasi-symmetrical way. On the other hand, the diagonal displacements do not apparently fulfill this hypothesis, but this phenomenon can be ascribed to local defects or inhomogeneities that alter the expected stress and strain diagonal patterns.

**Figure 14:**
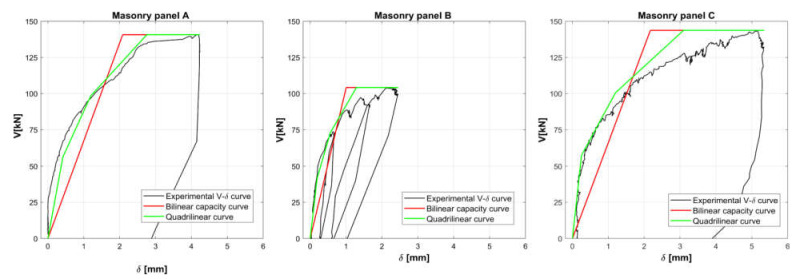
Experimental shear capacity curves of tested stone masonry walls (in black), bilinear approximation (in red), and quadrilinear curve (in green).

The resulting shear-displacement curves for the tested panels are shown in Figure 14, where, according to what remarked above, the shear stress in each subpanel V is assumed equal to 0.5 F and the horizontal displacement δ is considered the average value measured by the four central LVDTs (h.1, h.2, h.3, and h.4 in Figure 5).

Considering the actual compressive stress $\sigma_{0}$ provided by a single flat jack test (see Table 3), the shear strength $\tau_{k}$ can be evaluated from the maximum shear $V_{\max}$ by applying Equation (2). From the effective stiffness $k_{ef}$, already defined in Section 2.3 as secant stiffness at $0.7\;V_{\max}$ and evaluated considering the experimental V−δ in Figure 14, the shear modulus $G_{10-70}$ can be estimated by back-calculating Equation (4) and considering the elastic modulus $E_{10-70}$ previously derived from double flat jack tests_:_

(5) $$G_{10-70}=\frac{1.2\;k_{ef}\;E_{10-70}\;L^{2}\;h}{E_{10-70}\;L^{3}\;t-h^{3}\;k_{ef}}.$$

The experimental values of $V_{\max}$, $\tau_{k}$, $k_{ef}$, and $G_{10-70}$ obtained for each stone masonry panel are reported in Table 4.

The results confirm the high variability of stone masonry properties already highlighted in previous studies [6,7,16]. Looking at the ratio between shear modulus and shear strength, a typical range $G/\tau_{k}=1100\;–\;4000$ is assumed in [5,36]. Values of the ratio are in that typical range for panel A, for which $G/\tau_{k}=1570$, and panel C, for which $G/\tau_{k}=2897$, while a significantly higher value is obtained for panel B, $G/\tau_{k}=5611$.

Compressive strength $f_{m}$ and shear strength $\tau_{k}$ are well correlated: in fact, a linear relationship has been obtained:

(6) $$\tau_{k}=0.043\;f_{m}-0.029\;\mathrm{MPa},$$

with coefficient of determination $R^{2}=0.998$.

Notwithstanding the different strength and stiffness properties, a similar behavior can be noticed for the shear-deformation curves of the three masonry panels. The curves present

- a first quasi-elastic section, in the range $0.1\;V_{\max}-0.4\;V_{\max}$, characterized by very low displacements: the stiffness of this branch can be derived from Equation (4), assuming elastic and shear moduli, $E_{10-40}$ and $G_{10-40}$;
- a second section, in the range $0.4\;V_{\max}-0.7\;V_{\max}$, when first cracks occur in the masonry panels, characterized by higher displacements and by a consequent tangible reduction of the secant stiffness, which results in about 40% of the quasi-elastic one;
- a third section, in the range $0.7\;V_{\max}-V_{\max}$, characterized by a widespread crack pattern and a further reduced secant stiffness, which drops to about 20% of the quasi-elastic one.

In Figure 14, the three linear sections of the load-displacement curve are shown in green, together with the bilinear capacity curve (in red) and the experimental one (in black), while in green a further piecewise linear approximation (quadrilinear) is shown. It must be underlined that the quasi-elastic section can also be extended till $0.7\;V_{\max}$.

## 4. Discussion

The evaluation of the shear force-deformation behavior of stone masonry walls is a challenging issue [37] due to the high variability of masonry properties that are present in historical buildings. The preliminary results described in the previous section do not allow for drawing general conclusions, but a first comparison can be made with existing models and structural code provisions [19].

**Figure 15:**
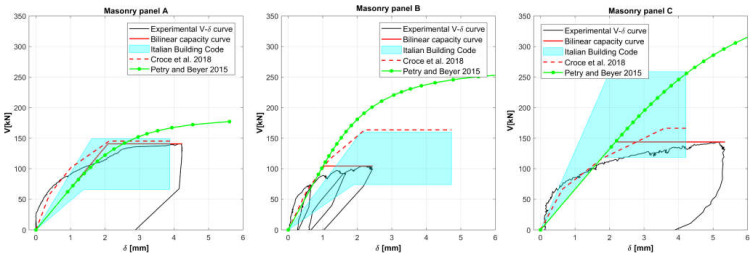
Shear response of tested masonry panels A, B, and C: experimental one (in black), equivalent bilinear capacity curve from the experimental one (solid red lines), possible range according to [19] (in cyan), capacity curves evaluated based on the procedure proposed in [7] (dashed red lines), and analytical model [38] (green lines).

The experimental curves obtained on the three stone masonry panels are compared with alternative capacity curves in Figure 15.

The diagrams in Figure 15 show:

- The bilinear curves obtained considering the range of parameters given for the “Partially Dressed Stone” and “Fully Dressed Stone” in [19], already reported in Table 1. Values of elastic and shear modulus are reduced by 50% to account for the influence of cracking [31], while correction factors for high-quality masonry are also considered to improve the evaluation of the maximum strength.
- The curves derived following the procedure proposed by the authors in [7], where the relevant parameters, G and $\tau_{k}$, are derived from the elastic modulus, using suitable relationships (i.e., G=0.15 E and $\tau_{k}=G/2000$). The rationale of the proposal is that the elastic modulus is the mechanical parameter that can be more easily and accurately measured by means of in situ tests, like double flat jack tests.
- The curves provided by the analytical model proposed by Petry and Beyer in [38] for the force-displacement response of an unreinforced masonry panel with a dominating flexural mode.

Looking at the diagrams, it clearly emerges that:

- Bilinear curves based on the wide range of parameters given by the guidelines for the application of the Italian Building Code [19] for stone masonry may still not cover experimental values.
- The procedure proposed in [7] well fits the experimental shear response for masonry panels A and C, while it overestimates the shear strength in the case of panel B, but this result depends on the exceptionally high $G/\tau_{k}$ ratio exhibited by panel B.
- The Petry and Beyer analytical model systematically overestimates the experimental force-displacement response and the shear strength. The overestimation, which is still acceptable for panel A, is clearly too large in the case of panels B and C, which are subjected to high normal stresses.

## 5. Conclusions

The evaluation of the shear behavior of stone masonry walls is a challenging task and a fundamental step for a reliable assessment of the seismic performance of existing masonry buildings.

In the paper, a combined in situ test procedure for the experimental characterization of masonry mechanical parameters is presented, together with the first results obtained during the experimental campaign carried out by the authors. The procedure, which consists of a combination of three different in situ tests (single flat jack test, double flat jack test, and shear compression test), provides a complete characterization of masonry properties, reducing the uncertainty in the estimation of the capacity curves of masonry walls and consequently on the evaluation of seismic performance. The innovative test arrangement is very promising since it reduces the invasiveness of the shear compression test with respect to common procedures, making also easier the repair and restoration activity after the test.

The preliminary results confirm the high variability of masonry properties. Indeed, a comparison with existing models and reference values for masonry properties demonstrates that the predicted shear response based on the simplified approach suggested in [7] satisfactorily fits the experimental behavior when Giotto and Niccolini building walls (panels A and C) are concerned, while it overestimates the shear response in the case of Cairoli’s wall (panel B), which is characterized by an exceptionally high value of the ratio $G/\tau_{k}$.

A satisfactory correlation is found between the experimental values of compressive and shear strength, but further experimental results are needed to draw more general conclusions and to confirm the validity of the obtained linear relationship. This will be one of the subjects of an ongoing experimental campaign.

## Figures and Tables

**Table 1:** Reference values of relevant properties of different types of masonry: compressive strength, $f_{m}$; shear strength, $\tau_{k}$; elastic modulus, E; shear modulus, G; and specific weight, w (adapted from Table C8. 5. I of Italian Guidelines 2019 [19]).

Masonry Type	$f_{m}\;(N/{\mathrm{mm}}^{2})$	$\tau_{k}\;(N/{\mathrm{mm}}^{2})$	$E\;(N/{\mathrm{mm}}^{2})$	$G\;(N/{\mathrm{mm}}^{2})$	$w\;(\mathrm{kN}/{\mathrm{mm}}^{3})$
Rubble or irregular stone	1.00–2.00	0.018–0.032	690–1050	230–350	19
Partially dressed stone	2.00	0.035–0.051	1020–1440	340–480	20
Fully dressed stone	2.6–3.8	0.056–0.074	1500–1980	500–660	21
Irregular soft stone	1.4–2.2	0.028–0.042	900–1260	300–420	13–16
Regular soft stone	2.0–3.2	0.04–0.08	1200–1620	400–500	13–16
Squared stone blocks	5.8–8.2	0.09–0.12	2400–3300	800–1100	22
Solid brick and lime mortar	2.6–4.3	0.05–0.13	1200–1800	400–600	18
Hollow bricks and cement mortar	5.0–8.0	0.08–0.17	3500–5600	875–1400	15

**Table 2:** Statistical parameters for stone masonry properties based on the collected database and the identified classes (based on data derived from [2]).

Masonry Property	Class	Mean (N/mm^2^)	COV
Compressive strength $f_{m}$	All data	1.88	0.33
	Class I	1.18	0.29
	Class II	1.91	0.12
	Class III	2.59	0.13
Elastic modulus $E_{10-40}$	All data	1816	0.39
	Class I	1164	0.20
	Class II	2088	0.15
	Class III	2857	0.08
Shear modulus $G_{10-40}$	All data	665	0.53
	Class I	429	0.32
	Class II	887	0.11
	Class III	1420	0.05

**Table 3:** Characteristics of the investigated stone masonry panels.

Masonry Panel	h (m)	L (mm)	t (mm)	$\sigma_{0}$ (N/mm^2^)	$f_{m}$ (N/mm^2^)	$E_{10-40}$	$E_{40-70}$	$E_{70-100}$
						(N/mm^2^)	(N/mm^2^)	(N/mm^2^)
A	970	900	480	0.465	3.56	3022	1455	807
B	1183	840	600	0.76	2.23	2767	1829	1113
C	1050	980	660	0.67	1.78	1543	1543	407

**Table 4:** Experimental values of the ultimate shear force $V_{\max}$, the shear strength $\tau_{k}$, the effective stiffness $k_{ef}$, and the corresponding shear modulus $G_{10-70}$.

Masonry Panel	$V_{max}$ (kN)	$\tau_{k}$ (N/mm^2^)	$k_{ef}$ (kN/mm)	$G_{10-70}$ (N/mm^2^)
A	140.8	0.126	67.6	198
B	104.3	0.066	103.7	369
C	143.8	0.050	66.2	145

## Data Availability

The data presented in this study are available on request from the corresponding author. The data are not publicly available as they cannot be used for commercial purposes.
